# Introducing an arterial non-injectable connector into clinical practice

**DOI:** 10.1186/cc13278

**Published:** 2014-03-17

**Authors:** D Laba, M Mariyaselvam, R Heij, P Young

**Affiliations:** 1 The Queen Elizabeth Hospital, Kings Lynn, UK

## Introduction

The standard arterial system for blood withdrawal provides no impediment to intra-arterial injection [1] and bacterial contamination. We assessed the probability of inadvertent intra-arterial injection, transmission of bacterial contamination and surveyed the nursing staff following introduction of a non-injectable connector (NIC).

## Methods

The simulation study data were descriptive. Fifteen junior doctors managed a case of bradycardia. A simulated patient had a peripheral intravenous cannula, central venous catheter and brachial arterial cannula. Atropine was available on request. A laboratory controlled trial compared a transmission of bacteria through standard arterial ports against the NIC when attached to an arterial sampling hub. The colonization rates were compared using a two-tailed Fisher's exact test. A closed-circuit arterial sampling system was designed. A contaminated syringe tip was inserted into the conventional arterial hub or the NIC to take an arterial sample. Transducer flush fluid flowing to the patient bloodstream was cultured. In a survey the nursing staff were asked questions regarding the NIC system, including its manual handling, sampling of the blood and its durability.

## Results

In the simulation study 10/15 clinicians (67%) injected atropine directly into arterial cannula via a three-way tap. Five of 15 doctors (34%) injected safely. In the laboratory study, swabbing of the arterial hubs showed bacterial contamination of all of the samples in the standard group (20/20) and no contamination in the NIC group (0/20), *P *< 0.0001. Eighty-five per cent (17/20) of samples in the standard arterial group showed onward transmission of pathogens to the patient circulation, and none of the patient samples taken from the NIC group were contaminated (0/20), *P *< 0.0001. In the survey, 80% of the nurses found manual handling of a new device equally simple and 20% even simpler. Sampling of the blood was equally challenging for 87% participants. Ninety-four per cent found similar durability of the new device and better protection against accidental intra-arterial injection. Sixty-six per cent of the nursing staff would replace the standard system with a NIC.

## Conclusion

Most of the doctors would inject the drug intra-arterially. Currently, no other device is available to eliminate accidental intra- arterial injection or bacterial transmission. The nursing staff found the management of the NIC equally challenging.

## References

[B1] Sen S Complications after unintentional intraarterial injection of drugs: risks, outcomes, and management strategies Mayo Clin Proc 2005 80 783 795 15945530 10.1016/S0025-6196(11)61533-4

